# Psychopathological Symptoms and Loneliness in Adult Internet Users: A Contemporary Public Health Concern

**DOI:** 10.3390/ijerph17030856

**Published:** 2020-01-30

**Authors:** Ângela Leite, Ana Ramires, Susana Amorim, Hélder Fernando Pedrosa e Sousa, Diogo Guedes Vidal, Maria Alzira Pimenta Dinis

**Affiliations:** 1 Faculdade de Filosofia e Ciências Sociais, Universidade Católica Portuguesa, Rua de Camões 60, 4710-362 Braga, Portugal; angelamtleite@gmail.com; 2 Faculdade de Turismo e Hospitalidade, Universidade Europeia, Rua Laura Ayres 4, 1650-510 Lisboa, Portugal; ana.ramires.ps@gmail.com; 3 Universidade Lusófona, Rua de Augusto Rosa 24, 4000-098 Porto, Portugal; amorim.suzy@gmail.com; 4 Department of Mathematics (DM. UTAD), University of Trás-os-Montes and Alto Douro, Quinta de Prados, 5001-801 Vila Real, Portugal; hfps@utad.pt; 5 UFP Energy, Environment and Health Research Unit (FP-ENAS), University Fernando Pessoa (UFP), Praça 9 de Abril 349, 4249-004 Porto, Portugal; madinis@ufp.edu.pt

**Keywords:** time spend on internet, problematic internet use, loneliness, psychopathological symptoms, well-being

## Abstract

There are different concepts that translate abusive Internet use. Almost all these concepts converge on excessive time spent online, which can trigger the emergence of problematic situations. Most of the studies reported in the literature, both nationally and internationally, focused on a young population and found negative consequences of this Internet misuse. The objective of this study consists of associating the time spent using the Internet—in years, times per week, and hours per day—with psychopathological symptoms, as well as assessing the perception of loneliness, in an adult Portuguese population. A quantitative approach, based on a survey application, was conducted in a convenience sample composed by 418 participants (64.4% female), with a mean age of 29.9 years old (*SD* = 9.26), ranging from 18 to 73 years. The results suggest that maladaptive patterns of Internet use found in young people seem to be replicated in the adult population. A relationship between time spent on the Internet and psychopathological symptoms, and an association between loneliness and the number of hours spent on the Internet, were also identified. In an individualized and disconnected offline world, Internet impact in individuals’ well-being results must be highlighted, since it should be understood as a public health issue. The novelty of this study lies in the target population: Portuguese Internet users over 18 years of age, for which there is no specific study on the subject, thus emphasizing the transverse nature of the problem.

## 1. Introduction

In a world dominated by the Internet, where almost 60% of the world population are presently Internet users [1], and in Portugal where almost 79% of the Portuguese population uses the Internet [2], the Internet is an integral part of everyday life, used for communicating, listening to music, shopping, reading, working, and learning online or using social networks [3]. Cyberspace assumes the role of a public space, in a virtual way, where individuals socialize and create their relationships with society. This is, according to Bauman [4], the “Liquid Love” or “Liquid Modernity” which means that at present, it is not about relationships, but about connections. In fact, many friendships are born on social networks and sometimes the real mixes with the virtual. When someone wants to make another feel upset, it is easy: The “delete” button is used to delete the connection or, most impressively, the relationship. The abusive use of the Internet can thus trigger the emergence of problematic situations and, most importantly, the impact the possibility to live one’s life.

The abusive use of the Internet is considered a behavioural addiction, in the form of financial (e.g., gambling and shopping), palatable food, sex-related (e.g., cybersex, intercourse, and pornography), and media (e.g., computer, Internet and video game) addictions. Internet addiction disorder [5], Internet addiction [6,7], computer addiction [8], problematic Internet use [9], pathological Internet use [10,11], compulsive Internet use [12], and impulsive-compulsive Internet usage disorder [13] are different concepts that translate approximate realities. Almost all of these concepts converge on excessive time spent online and are related to negative consequences [14,15,16,17]. However, at a time when the majority of people need to use Internet to work or study, it becomes difficult to separate time spent on activities to fulfil professional commitments from the time spent to satisfy a need that cannot be controlled: Society is the creator of both necessities—not basics but, rather indispensable for being “connected” and part of it—and the space to satisfy these needs. The example of this situation is visible in studies focusing on the phenomenon known as the digital divide. It is, in fact, a problem, not only due to the unequal distribution of the access to the Internet or technology, but also because it results in the exclusion from the information society or digital society [18]. 

Furthermore, will the problem be the use of the Internet itself, or is that use just a new manifestation of something already known? According to Volpe and colleagues [3] “some specific entities can be seen as old psychopathological phenomena that have been reconfigured by new technologies, others are so intrinsically linked to cyberspace that (…) are considered as new problems born out of a new type of interaction between humans and technology”, namely online gambling, Internet gaming disorder, cyberchondria, cybersuicide, cybersex, cyberbullying/cyberstalking, compulsive online shopping, and other Internet-related psychopathologies. The presence of other psychiatric conditions in patients with problematic Internet use is the rule rather than the exception [19]. Abusive Internet use may imply a behavioural risk syndrome and a clinical disorder, regarding the presence of withdrawal and tolerance symptoms [20]. Depression and symptoms of attention deficit and hyperactivity disorders have significant correlations with problematic Internet use and this association was reported to be higher among males [15,21], although Twenge and colleagues [21] found that depression and suicidal rates increase more in adolescent girls than in adolescent boys.

The pathway from adaptive Internet use to pathological Internet use is not clear, although it is possible to distinguish those two kinds of Internet utilization [15]. Three patterns of Internet use, i.e., ‘social’, ‘sex and games’, and ‘functional’, were identified by Reiner and colleagues [22], although the authors do not completely agree with which of these patterns are adaptive and which are non-adaptive. Adaptive use includes keeping old friendships and making new ones, promoting socialization, reducing loneliness [15], the high use of research, emails and Internet shopping [22], while non-adaptive Internet use implies spending more hours online carrying out specific activities [15], and social Internet use (for boys and girls) [22]. 

Kuss and colleagues [23] found that dysfunctional coping strategies, i.e., distraction, denial, self-blame, substance use, venting, media use, and behavioural disengagement can help in predicting excessive Internet use; and media-focused coping and substance use coping mediate the relationships between psychopathology and excessive Internet use. Kaess and colleagues [24] also found that psychopathology and suicidal behaviours are strongly related to problematic Internet use, with this association being significantly conditioned by gender and country, suggesting socio-cultural influences. 

According to Widyanto and Griffiths [25], empirical research about this issue covers five areas: Comparisons of excessive Internet users and non-excessive Internet users, the study of groups that are vulnerable to excessive Internet use, the examination of the psychometric properties of excessive Internet use, the study of Internet users and treatment case studies, and the study of the relationship between excessive Internet use and other behaviours.

## 2. Materials and Methods 

### 2.1. Objectives and Hypotheses

The objective of the present study is included in the latest area of research, i.e., studying the connection between excessive Internet use and other behaviours, consisting of relating the time spent using the Internet—measured in years, times per week, and hours per day—with psychopathological symptoms, as well as with the perception of loneliness. The authors’ hypothesis is that, in the light of the scientific literature available, people who spend more time on the Internet present more psychopathological symptoms and experience more loneliness. The novelty of this study lies in the target population, i.e., Portuguese users over 18 years of age, for which there is no specific study on the subject until now, except for the validation of scales assessing Internet addiction [26] and generalized problematic Internet use [27].

### 2.2. Procedures

All procedures were carried out in accordance with the Declaration of Helsinki. Participants were informed of the study’s objectives and were given the guarantee of anonymity and of the confidentiality of the data, after which they gave their written consent. Participants understood the need for such studies to prevent maladaptive behaviours and the authors compromised to share the results of the research. The protocol of this study was approved by the Ethics Committee of the Portuguese Catholic University, internal reference number—PCU/2018/02/PT. The inclusion criteria comprised being 18 years of age or older, Portuguese, and having access to the Internet at home. The protocol was applied to 418 participants of the general population, obtained from a convenience sample. The application of the protocol took place in varied contexts, namely, universities, health centres, and shopping centres. Institutional authorizations were requested and obtained for the application of the questionnaires in the different contexts and facilities. The participants were contacted by the researchers in the abovementioned contexts, and those who voluntarily decided to participate answered the questionnaire in a room previously allowed for this purpose in different contexts. 

### 2.3. Measures

The applied protocol contained sociodemographic questions; three questions related to Internet use time, and four instruments assessed psychopathological symptoms, depression, anxiety, and loneliness. In relation to sociodemographic questions, data were collected on: Gender; age, categorized as ≤ 22 (late adolescence), 23–29 (emerging adulthood), 30–36 (young adulthood), and ≥ 37 (middle adulthood until seniority) years old; relationship status, i.e., categorized as not in an affective relationship (single, divorced, separated, and/or widowed) and in an affective relationship (boyfriend/girlfriend, married, or in an unmarried union); having children, for which the answer choices were yes or no; education, categorized as having a higher education degree (BSc, MSc, and PhD) or not having a higher education degree (elementary and secondary); professional situation (active or inactive); having hobbies; exercising; considering themselves to eat properly; and considering themselves to be overweight (yes or no). Questions related to the time of Internet use were: “How many years have you been using Internet?”, categorized into four options, ≤4, 5–10, 11–15, and >15 years; “Do you use Internet daily?” (yes or no); and “How many hours per day do you use Internet?”, categorized as ≤1, 2–4, and >4 hours. The four instruments evaluating psychopathological symptoms, depression, anxiety, and loneliness are described below:

*Psychopathological symptoms*: Brief Symptom Inventory (BSI) [28]. BSI is a 53-item self-report instrument that assesses psychological symptoms. It is composed of nine primary symptom dimensions: Somatization, obsessive-compulsive, interpersonal sensitivity, depression, anxiety, hostility, phobic anxiety, paranoid ideation, and psychoticism; and three global indices of distress: Global Severity Index (GSI), Positive Symptom Total (PST) and Positive Symptom Distress Index (PSDI), which measure the overall psychological distress level, the number of self-reported symptoms, and the intensity of symptoms. Each item is answered on a 5-point Likert scale of frequency (from 0—never, to 4—always). Rankings characterize the intensity of distress during the past seven days.

*Depression*: The Beck Depression Inventory (BDI) [29,30]. BSI is a 21-item self-report rating inventory that measures characteristic attitudes and symptoms of depression. Participants report the intensity of depressive symptoms on a scale from zero (absence of symptomatology) to three (severe symptomatology), according to how they felt during the last week, obtaining a total score ranging from 0 to 63. It also allows the intensity of depressive symptomatology to be categorized as follows: 0–9, non-depressed; 10–16, dysphoria; 17–20, mild depressive states; 21–30, moderate depression; and >30, severe depression [30].

*Anxiety:* Self-Rating Anxiety Scale (SAS) [31] Zung [32]. SAS is a 20-item scale, in which items relating psychological and physiological symptoms are rated by respondents according to the past week, using a 4-point Likert scale ranging from one (none or a little of the time) to four (most or all the time). The scoring is based on four groups of symptoms: Cognitive—thinking, understanding, learning, remembering; autonomic—the involuntary part of the nervous system; motor—body movement; central nervous system (CNS)—brain and spinal cord. The score ranges from 20 to 44—normal; 45 to 59—mild to moderate anxiety levels; 60 to 74—marked to severe anxiety levels; 75 to 80—extreme anxiety levels.

*Loneliness*: The UCLA Loneliness Scale (UCLA) [33,34] consists of 18 items assessing loneliness as well as the feelings underlying it. Half of the items were inversely formulated. The answers of each item are made in a Likert model scale with 5 modalities, from never (1) to forever (5). The scale was used to obtain a general score, revealing good psychometric qualities.

### 2.4. Statistical Analyses

Statistical analyses comprised: (i) Univariate analysis to characterize the sample; (ii) normality tests and heterogeneity tests for assessment of data distribution; (iii) student’s *t*-tests for the independent samples, and one-way ANOVA tests with Games–Howell post-hoc test for mean comparisons of psychopathological symptoms, depression, anxiety, and loneliness according to sociodemographic and Internet use variables. To assess the internal consistency of the scales, Cronbach’s alpha was applied. The Cohen’s *d* effect size of the comparisons was calculated. The tested hypothesis, i.e., people who spend more time on the Internet present more psychopathological symptoms and experience more loneliness, was tested for a significance level of 0.05. SPSS 24.0 package program ((IBM, Armonk, NY, USA)) was used to perform statistical analysis.

## 3. Results

The sample was composed mostly of female participants. The mean age is 29.87 years old (*SD* = 9.26), ranging from 18 to 73 years. Half of the sample (*n* = 210) were not in an affective relationship (single, divorced, separated, and/or widowed). The majority of the sample did not have children, had a university degree (*n* = 224), and were professionally active. Also, the majority had hobbies, exercised, consider themselves to eat well, and were not overweight (Table 1). 

Most of the participants had been using the Internet for 5–15 years, daily, and about 2–4 hours per day (Table 2).

All scales and subscales, with the exception of the “Central nervous system” subscale of the Self-Rating Anxiety Scale, present α > 0.5 (Table 3).

Psychopathological symptoms, depression, anxiety, and loneliness were compared according to sociodemographic variables. Only statistically significant results are presented. 

### 3.1. Gender

Women presented significantly higher values than men in almost all studied variables (Table 4).

### 3.2. Age

In relation to age (categories ≤22, 23–29, 30–36, and ≥37 years), younger participants, i.e., under 23 years, were found to have significantly (*p* = 0.023) higher values than the older ones (≥37), with regard to obsessive compulsive dimensions and motor anxiety (*p* = 0.032) (Table 5).

### 3.3. Relationship Status

Participants who were not in an affective relationship were found to present significantly higher psychopathology values than those who were in an affective relationship (Table 6). 

### 3.4. Having Children

Those who had children were found to have significantly higher depression (somatic and performance) values than those who did not have children.

*Depression (BDI):* Somatic and Performance [0.41 ± 0.40 versus 0.30 ± 0.32; *t*(145, 77) = 2.48, *p* = 0.014, *d* = 0.32].

### 3.5. Education

Participants without a higher education degree presented significantly higher values than those with a higher education degree in relation to psychopathology and depression (Table 7).

### 3.6. Professional Situation

Professionally active participants presented significantly higher values than the inactive ones, only relating to somatic and performance depression.

*Depression (BDI):* Somatic and Performance [0.36 ± 0.34 versus 0.28 ± 0.33; *t*(416) = 2.17, *p* = 0.030, *d* = 0.22].

### 3.7. Hobbies

Participants without hobbies presented higher values than the subjects with hobbies, with regard to psychopathology, depression, and anxiety (Table 8).

### 3.8. Exercise

Participants who did not exercise had significantly higher psychopathology, depression, and anxiety values than those who did exercise (Table 9). 

### 3.9. Eating Habits

Participants who did not consider themselves to be well fed presented significantly higher values than those who considered that they eat well, with regard to psychopathology, depression, anxiety, and loneliness (Table 10).

### 3.10. Weight

Participants who considered themselves to be overweight revealed significantly higher values than those who considered that they were not overweight, with regard to psychopathology, anxiety, and loneliness (Table 11). 

The means of the dependent variables according to Internet use were compared. In Table 12, Table 13 and Table 14, only statistically significant results are presented. The “5–10 years” Internet users showed significantly higher mean values in: Some psychopathological symptoms—total, somatization, GSI and PST—than users who do it over 11 years; obsessive-compulsive symptoms compared to those who had been using Internet for the longest time (>15 years); motor anxiety symptoms compared to the “11–15 years” Internet users; anxiety and PSDI symptoms compared to “<5 years” Internet users. Daily Internet users had higher mean depression levels—total and cognitive affective—than those who did not use Internet daily. Participants who spent at least two hours per day revealed higher mean levels of loneliness than those who spent up to one hour per day. 

One in three of the participants in the study manifested psychopathological symptoms, one in four presented depression to different degrees and symptoms of loneliness; and only one in ten manifested anxiety (Table 15).

## 4. Discussion

One possible reason for the increase of mental health problems is the growing utilization of virtual communication [21]. Internet-related psychopathology represents a new challenge to well-being [3]. As time spent using the Internet is one of the best predictors of its misuse [14,15], it is intended to determine whether people who spent the most time using the Internet had higher values of psychopathology, depression, anxiety, and loneliness than those who spent less time on the Internet, not taking into consideration whether time would be excessive or not. As Durkheim has stated [35], more than ever, contemporary society is experiencing a new way of “anomie”, visible in the distrust in the classic structures, i.e., family, religion, friends, and in the identification with ephemeral virtual institutions, which is in part related to Bauman [4] theory.

In this study, time was measured accounting for the number of years of Internet use, considering a daily use (or not), and the number of hours of use per day. Twenge and colleagues [21] had already suggested that the results of using electronic communication depended, among other aspects, on the time spent in this activity. Although not aiming to establish a direct association between time spent on the Internet and its problematic use, that cannot be fully achieved because it is the only indicator of this study in relation to Internet use.

Regarding years of Internet use, it was found that one of the intermediate groups, i.e., 5–10 years ago, was the one with the highest values of psychopathological symptoms, i.e., somatization, obsessive-compulsive, anxiety, psychoticism, GSI, PST, and PSDI, in relation to the other three groups; this was the group presenting the closest value (1.67) of the BSI cut-off point suggested by the authors of the Portuguese version (1.7) [28]. However, this was not the group that had been using the Internet for the most years, since the two other groups had been using the Internet for 11–15 years and over 15 years; hence, the hypothesis that these two last groups should present worse values than the other groups, was not confirmed. Although this study did not control the type of activity carried out by Internet users during its use, it may be plausible to conclude that people who have been using the Internet for more years did it for professional reasons; 10 years ago, Internet popularity was not the same as today. As already observed, Internet use for work purposes is considered functional and adaptive [15,22], which may justify why these participants do not present higher values in the assessed psychopathological dimensions. It may be also possible to question whether the measure “years” is adequate for quantifying the time spent on the Internet, since this unit of time may just be related to the age of the participants and not to a (large) amount of years of Internet use.

In agreement with the results presented in this text, Taymur and colleagues [36] have also found that somatization increased with the severity of Internet addiction. In the systematic review on the association between pathological Internet use and comorbid psychopathology, Carli and colleagues [15] found five studies on the relationship between problematic Internet use and obsessive-compulsive symptoms, of which three reported full associations and other partial associations only for men. Andreassen et al. [37] found that obsessive-compulsive disorder was positively related to addictive use of social media. Regarding anxiety, Carli and colleagues [15] also mention the existence of 7 studies, 4 of which report a total association between anxiety and Internet use and 3 do not report any association. According to Kuss and Lopez-Fernandez [5], anxiety disorders appear to be particularly common in the problematic use of the Internet. Nakayama, Mihara, and Higuchi [38] reported psychotic symptoms and high psychoticism scores in problematic Internet users. Montag and Reuter [39] suggested that psychoticism may represent the best predictor for (generalized) Internet addiction. Although the participants that used Internet for more years did not present statistically significant differences regarding aggressiveness, as compared to participants with less years of Internet use, participants with 5–10 years of Internet use had higher values of motor anxiety and, as Lim and colleagues [40] argue, it may be predisposing to aggressive behaviour. Kuss et al. [23] also found a relationship between GSI and excessive Internet use.

Subjects who used the Internet daily also had higher cognitive-affective depression values than those who did not use the Internet daily. This result implies a reflection on the quality of virtual connections. Using Internet daily and being connected with the virtual dimension does not result in socialization gains. On the contrary, it results in a feeling of emptiness which could lead to more vulnerability to depression. Banjanin, Banjanin, Dimitrijevic, and Pantic [41] showed that Internet use and Internet addiction are positively correlated with depressive symptoms. Moreover, Katikalapudi, Chellappan, Montgomery, Wunsch, and Lutzen [42] found that students with depressive symptoms used the Internet much more than those without symptoms. Romano, Osborne, Truzoli, and Reed [43] stated that high problem Internet users showed a pronounced decrease in mood following Internet use compared to the low problem Internet users. Twenge and colleagues [21] stated that adolescents who spent more time on new media activities presented increases in depression and suicide. The studies mentioned here were mostly performed with adolescent and young samples, which was not the case in the present study. In this study, no significant differences were found regarding depression in relation to age groups.

Subjects who used the Internet more than two hours a day showed more loneliness than those who used it less time per day, which may not be problematic, as Carli and colleagues [15] consider that a functional use may aim to combat and reduce loneliness. Taylor, Pattara-angkoon, Sirirat, and Woods [44] stated that all subtypes of Internet addiction share some characteristics, one of them being tolerance, with individuals spending an increasing number of hours online to achieve the same level of satisfaction. Tonioni and colleagues [45] considered that a misuse of Internet is characterized by many hours spent online. Dhaka and colleagues [46] found differences in hours spent per day for females (4.5 h) and males (6.4 h), contrary to the data in this study, in which most men and women use the Internet between 2 and 4 hours per day. Also, Shen, Liu, and Wang [47] reported that problematic Internet use increases social and psychological problems such as loneliness and social isolation. Morahan-Martin and Schumacher [48] consider that lonely individuals used the Internet and email more and were more likely to use the Internet for emotional support than others.

One in three of the participants in our study manifested psychopathological symptoms, one in four presented depression to different degrees and symptoms of loneliness, and only one in ten showed anxiety. These results corroborate previous studies on the use of the Internet and its relationship with psychopathological symptoms [15,19,21]. The only studies with a Portuguese population, carried out by Pontes and colleagues [26,27], classified the participants in three classes: Low risk (*n* = 289, 46.7%), medium risk (*n* = 256, 40.7%), and high risk (*n* = 77, 12.6%) of problematic Internet use, with the prevalence of Internet addiction being 1.2%. The higher risk sample was a similar size to the sample in this study with higher values of psychopathology.

## 5. Conclusions

Maladaptive patterns of Internet use found in young people seem to be replicated in the adult population, as the results of this study have shown. A relationship between time spent on the Internet and psychopathological symptoms, and an association between loneliness and number of hours spent on the Internet, were also identified. In an individualized and disconnected offline world, results about the Internet’s impact on individuals’ well-being must be highlighted, since it should be understood as a public health issue. The novelty of this study lies in the target population: Portuguese Internet users over 18 years of age, for which there is no specific study on the subject, thus emphasizing the transverse nature of the problem. As emphasized before, most of the studies reported in the literature, both nationally and internationally, focused on young populations, in contrast to the sample used in this study. However, the results were overlapping, which may suggest that the maladaptive patterns of Internet use found in young people seem to be replicated in an adult population, and this fact must be highlighted and explored in further and future research.

## Figures and Tables

**Table 1:** Profile of the respondents.

Variable	*n*	%
**Gender**		
Male	149	35.6
Female	269	64.4
**Age Categories**		
≤ 22	108	22.8
23–29	118	28.2
30–36	105	25.1
≥37	87	20.8
**Marital Status**		
Single	179	42.8
Dating	90	21.5
Married/living together	118	28.2
Divorced/separated	30	7.20
Widow	1	0.20
**Children**		
No	316	75.6
Yes	102	24.4
**Education**		
Elementary	30	7.20
Secondary	164	39.2
BSc	153	36.6
MSc	66	15.8
PhD	5	1.20
**Professional situation**		
Non-active	163	39.0
Active	255	61.0
**Hobbies**		
No	154	36.8
Yes	264	63.2
**Exercise**		
No	170	40.7
Yes	248	59.3
**Eat properly**		
No	97	23.2
Yes	321	76.8
**Overweight**		
No	321	76.8
Yes	97	23.2

**Table 2:** Internet usage profile.

	*n*	%
**Years of Internet use**		
≤4	30	7.18
5–10	199	47.6
11–15	148	35.4
>15	41	9.81
**Daily Internet use**		
No	67	16.0
Yes	351	83.9
**Hours per day of Internet use**		
≤1	137	32.8
2–4	208	49.8
>4	73	17.5

**Table 3:** Alpha Cronbach (α) results for scales and subscales.

Scale	Subscale	*α*	Items
Brief Symptom Inventory (BSI)		0.967	53
	Somatization	0.853	6
	Obsessive-compulsive	0.759	6
	Interpersonal sensitivity	0.780	4
	Depression	0.876	6
	Anxiety	0.839	6
	Hostility	0.807	5
	Phobic anxiety	0.766	5
	Paranoid ideation	0.795	5
	Psychoticism	0.732	5
Beck Depression Inventory (BDI)		0.879	21
	Cognitive	0.839	9
	Somatic	0.774	12
Self-Rating Anxiety Scale (SAS)		0.796	20
	Cognitive	0.665	5
	Autonomic	0.552	9
	Motor	0.534	4
	Central nervous system	0.098	2
UCLA Loneliness Scale (UCLA)		0.832	18

**Table 4:** Significant means differences of gender.

Psychological Variables	Men		Women				
	*M*	*SD*	*M*	*SD*	*t* (df)	*p*	*d*
BSI Total	41.81	29.40	51.94	33.30	3.11(416)	0.002	0.32
BSI Somatization	0.42	0.55	0.74	0.76	5.04 (386, 98)	0.001	0.47
BSI Obsessive-compulsive	1.03	0.66	1.24	0.70	2.97 (416)	0.003	0.30
BSI Interpersonal sensitivity	0.82	0.74	1.02	0.76	2.54 (416)	0.011	0.26
BSI Depression	0.86	0.77	1.03	0.82	2.13 (416)	0.034	0.22
BSI Anxiety	0.72	0.61	1.07	0.81	5.02 (378, 36)	<0.001	0.47
BSI Phobic anxiety	0.37	0.53	0.53	0.62	2.85 (348, 65)	0.005	0.28
BSI GSI	0.79	0.55	0.98	0.63	3.10 (416)	0.002	0.32
BSI PST	25.68	12.85	29.77	13.33	3.04 (416)	0.003	0.31
BSI PSDI	1.53	0.47	1.65	0.51	2.43 (327, 32)	0.016	0.24
BDI Total	5.52	6.30	6.99	7.09	2.10 (416)	0.036	0.21
BDI Somatic/performance	0.26	0.31	0.37	0.35	3.06 (340, 08)	0.002	0.30
SAS Total	32.07	6.52	35.25	7.88	4.42 (355, 62)	<0.001	0.43
SAS Cognitive	1.56	0.46	1.75	0.54	3.67 (347, 02)	<0.001	0.36
SAS Motor	1.59	0.50	1.79	0.55	3.59 (416)	<0.001	0.37
SAS Vegetative	1.56	0.33	1.69	0.41	3.44 (363, 98)	0.001	0.33
SAS CNS	1.90	0.66	2.07	0.71	2.42 (416)	0.016	0.25

Notes: BSI - Brief Symptom Inventory; GSI - Global Severity Index; PST - Positive Symptom Total; PSDI - Positive Symptom Distress Index; BDI - Beck Depression Inventory; SAS - Self-Rating Anxiety Scale; CNS - Central Nervous System; t = Student’s t-test; p = p-value; d = Cohen’s d.

**Table 5:** Significant means differences of age.

Psychological Variables	≤22		23–29		30–36		≥37				
	*M*	*SD*	*M*	*SD*	*M*	*SD*	*M*	*SD*	*F* (df)	*p*	*η* ^2^
BSI Obsessive-compulsive	1.36	0.75	1.13	0.61	1.11	0.68	1.06	0.70	3.80 (3, 414)	0.010	0.27
BSI Phobic anxiety	0.61	0.63	0.44	0.62	0.40	0.61	0.43	0.46	2.70 (3, 414)	0.045	0.01
SAS Motor	1.83	0.59	1.75	0.56	1.65	0.51	1.62	0.46	3.13 (3, 414)	0.026	0.02

Notes: BSI - Brief Symptom Inventory; SAS - Self-Rating Anxiety Scale; *F* = ANOVA *F* test; *p* = *p*-value; *η*^2^ = Eta squared.

**Table 6:** Significant means differences of relationship status.

Psychological Variables	Not in a Relationship		In a Relationship				
	*M*	*SD*	*M*	*SD*	*t* (df)	*p*	*d*
BSI Obsessive-compulsive	1.24	0.58	1.09	0.68	2.28 (416)	0.023	0.22
BSI Interpersonal sensitivity	1.03	0.74	0.86	0.77	2.29 (416)	0.022	0.22
BSI Depression	1.07	0.81	0.87	0.79	2.58 (416)	0.010	0.25
BSI Psychoticism	0.86	0.70	0.71	0.69	2.26 (416)	0.024	0.22
BSI PST	29.73	13.18	26.88	13.28	2.20 (416)	0.029	0.22

Notes: BSI - Brief Symptom Inventory; PST - Positive Symptom Total; *t* = Student’s *t*-test; *p* = *p*-value; *d* = Cohen’s *d.*

**Table 7:** Significant means differences of education.

Psychological Variables	Until the 12^th^ Year of Schooling		Degree or More				
	*M*	*SD*	*M*	*SD*	*t* (df)	*p*	*d*
BSI Total	52.07	33.48	45.09	30.94	2.21 (416)	0.027	0.22
BSI Hostility	1.10	0.82	0.87	0.69	3.08 (416)	0.002	0.30
BSI Phobic anxiety	0.54	0.63	0.41	0.55	2.25 (416)	0.025	0.22
BSI Paranoid ideation	1.39	0.80	1.14	0.83	3.21 (416)	0.001	0.32
BSI Psychoticism	0.86	0.74	0.71	0.65	2.25 (416)	0.025	0.22
BSI GSI	0.98	0.63	0.85	0.58	2.22 (416)	0.027	0.22
BSI PST	29.96	12.46	26.88	13.84	2.39 (415, 36)	0.017	0.23
BSI PSDI	1.64	0.54	1.58	9.45	2.22 (416)	0.027	0.13
BDI Total	7.22	7.37	5.81	6.30	2.09 (382, 33)	0.037	0.21
SAS Cognitive	0.34	0.40	0.26	0.33	2.15 (377, 62)	0.032	0.21

Notes: BSI - Brief Symptom Inventory; GSI - Global Severity Index; PST - Positive Symptom Total; PSDI - Positive Symptom Distress Index; BDI - Beck Depression Inventory; SAS - Self-Rating Anxiety Scale; *t* = Student’s *t*-test; *p* = *p*-value; *d* = Cohen’s *d*.

**Table 8:** Significant means differences of hobbies.

Psychological Variables	No Hobbies		Hobbies				
	*M*	*SD*	*M*	*SD*	*t* (df)	*p*	*d*
BSI Total	54.90	32.52	44.50	31.48	3.21 (416)	0.001	0.33
BSI Obsessive-compulsive	1.29	0.70	1.09	0.67	2.87 (416)	0.004	0.29
BSI Interpersonal sensitivity	1.06	0.75	0.88	0.76	2.35 (416)	0.019	0.24
BSI Depression	1.10	0.81	0.89	0.79	2.60 (416)	0.010	0.26
BSI Anxiety	1.05	0.78	0.89	0.75	2.18 (416)	0.030	0.22
BSI Hostility	1.15	0.83	0.88	0.70	3.39 (279, 85)	0.001	0.36
BSI Phobic anxiety	0.58	0.60	0.41	0.58	2.89 (416)	0.004	0.29
BSI Psychoticism	0.94	0.76	0.69	0.64	3.62 (416)	<0.001	0.36
BSI PST	31.55	12.05	26.42	13.63	4.00 (352, 36)	<0.001	0.39
BSI PSDI	1.04	0.62	0.84	0.59	3.29 (416)	0.001	0.33
BDI Total	7.90	7.79	5.63	6.09	3.12 (262, 29)	0.002	0.34
BDI Cognitive	0.37	0.41	0.25	0.33	3.05 (263, 43)	0.002	0.33
BDI Somatic / performance	0.39	0.37	0.30	0.32	2.54 (280, 10)	0.011	0.27
SAS Motor	1.81	0.59	1.66	0.50	2.53 (277, 98)	0.012	0.27

Notes: BSI - Brief Symptom Inventory; GSI - Global Severity Index; PST - Positive Symptom Total; PSDI - Positive Symptom Distress Index; BDI - Beck Depression Inventory; SAS - Self-Rating Anxiety Scale; *t* = Student’s *t*-test; *p* = *p*-value; *d* = Cohen’s *d*.

**Table 9:** Significant means differences of exercise.

Psychological Variables	No Exercise		Exercise				
	*M*	*SD*	*M*	*SD*	*t* (df)	*p*	*d*
BSI Total	53.64	31.52	44.68	32.37	2.81 (416)	0.005	0.28
BSI Somatization	0.73	0.77	0.55	0.66	2. 44 (323, 64)	0.015	0.25
BSI Obsessive-compulsive	1.28	0.68	1.09	0.69	2.74 (416)	0.006	0.27
BSI Interpersonal sensitivity	1.08	0.74	0.86	0.76	2.96 (416)	0.003	0.30
BSI Depression	1.06	0.80	0.90	0.80	2.03 (416)	0.043	0.20
BSI Anxiety	1.07	0.82	0.86	0.72	2.78 (416)	0.006	0.28
BSI Hostility	1.10	0.79	0.89	0.73	2.79 (416)	0.005	0.28
BSI Phobic anxiety	0.55	0.61	0.41	0.58	2.37 (416)	0.018	0.24
BSI Psychoticism	0.88	0.72	0.71	0.67	2.49 (416)	0.013	0.25
BSI GSI	1.01	0.59	0.84	0.61	2.81 (416)	0.005	0.28
BSI PST	30.91	12.48	26.54	13.56	3.34 (416)	0.001	0.33
BSI PSDI	1.66	0.49	1.57	0.49	1.99 (416)	0.048	0.20
BDI Total	7.47	7.46	5.77	6.32	2.50 (416)	0.013	0.25
BDI Cognitive	0.35	0.40	0.26	0.34	2.28 (416)	0.023	0.23
BDI Somatic / performance	0.38	0.37	0.30	0.32	2.32 (416)	0.021	0.23
SAS Total	35.25	7.68	33.33	7.41	2.55 (416)	0.011	0.25
SAS Cognitive	1.76	0.52	1.63	0.51	2.54 (416)	0.011	0.25
SAS Motor	1.82	0.57	1.65	0.51	3.12 (416)	0.002	0.31
SAS CNS	2.09	0.73	1.95	0.67	1.99 (416)	0.047	0.20

Notes: BSI - Brief Symptom Inventory; GSI - Global Severity Index; PST - Positive Symptom Total; PSDI - Positive Symptom Distress Index; BDI - Beck Depression Inventory; SAS - Self-Rating Anxiety Scale; CNS - Central Nervous System; *t* = Student’s *t*-test; *p* = *p*-value; *d* = Cohen’s *d*.

**Table 10:** Significant means differences of healthy eating habits.

Psychological Variables	No Healthy Habits		Healthy Habits				
	*M*	*SD*	*M*	*SD*	*t* (df)	*p*	*d*
BSI Total	59.64	38.13	44.91	29.54	3.50 (132, 66)	0.001	0.47
BSI Somatization	0.77	0.86	0.58	0.65	2.03 (131, 31)	0.045	0.27
BSI Obsessive-compulsive	1.43	0.80	1.09	0.64	3.89 (135, 03)	<0.001	0.51
BSI Interpersonal sensitivity	1.15	0.90	0.89	0.70	2.64 (132, 92)	0.009	0.35
BSI Depression	1.25	0.90	0.88	0.76	3.63 (139, 85)	<0.001	0.46
BSI Anxiety	1.13	0.90	0.89	0.71	2.43 (134, 25)	0.017	0.32
BSI Hostility	1.20	0.91	0.91	0.70	2.80 (131, 94)	0.006	0.24
BSI Phobic anxiety	0.61	0.74	0.43	0.53	2.32 (127, 85)	0.022	0.32
BSI Paranoid ideation	1.46	0.91	1.20	0.79	2.84 (416)	0.005	0.33
BSI Psychoticism	1.05	0.78	0.70	0.65	4.43 (416)	<0.001	0.51
BSI GSI	1.13	0.72	0.85	0.56	3.50 (132, 64)	0.001	0.47
BSI PST	31.36	13.16	27.39	13.21	2.59 (416)	0.010	0.30
BSI PSDI	1.79	0.57	1.55	0.45	3.84 (134, 42)	<0.001	0.50
BDI Total	8.90	8.00	5.73	6.29	3.58 (133, 68)	<0.001	0.47
BDI Cognitive	0.42	0.42	0.26	0.34	3.36 (135, 80)	0.001	0.44
BDI Somatic / performance	0.44	0.40	0.30	0.32	3.22 (134. 74)	0.002	0.42
SAS Total	35.82	8.20	33.60	7.30	2.56 (416)	0.011	0.30
SAS Motor	1.86	0.61	1.68	0.51	2.64 (139, 23)	0.009	0.34
UCLA Total	40.32	8.69	37.97	9.20	2.23 (416)	0.026	0.26

Notes: BSI - Brief Symptom Inventory; GSI - Global Severity Index; PST - Positive Symptom Total; PSDI - Positive Symptom Distress Index; BDI - Beck Depression Inventory; SAS - Self-Rating Anxiety Scale; UCLA - UCLA Loneliness Scale; *t* = Student’s *t*-test; *p* = *p*-value; *d* = Cohen’s *d*.

**Table 11:** Significant means differences of weight.

Psychological Variables	Overweight		Not Overweight				
	*M*	*SD*	*M*	*SD*	*t* (df)	*p*	*d*
BSI Interpersonal sensitivity	1.11	0.89	0.90	0.71	2.07 (134, 24)	0.040	0.27
BSI PSDI	1.70	0.56	1.58	0.47	2.10 (416)	0.036	0.24
BDI Total	35.98	7.52	33.55	7.50	2.79 (416)	0.005	0.32
BDI Motor	1.83	0.53	1.68	0.54	2.40 (416)	0.017	0.28
BDI Vegetative	1.75	0.42	1.61	0.37	3.17 (416)	0.002	0.37
UCLA Total	41.16	8.01	37.71	9.30	3.58 (181, 21)	<0.001	0.38

Notes: BSI - Brief Symptom Inventory; PSDI - Positive Symptom Distress Index; BDI - Beck Depression Inventory; UCLA - UCLA Loneliness Scale; *t* = Student’s *t*-test; *p* = *p*-value; *d* = Cohen’s *d*.

**Table 12:** Significant means differences of dependent variables in relation to years of Internet use.

Variables	Years of Internet Use								One-Way ANOVA				Games–Howell Test
	≤4 (*n* = 30)		5–10 (*n* = 199)		11–15 (*n* = 148)		>15 (*n* = 41)						
	*M*	*SD*	*M*	*SD*	*M*	*SD*	*M*	*SD*	*F*	*df*	*p*	*η* *^2^*	
**BSI**													
Total	42.9	23.1	54.2	34.2	43.9	31.0	40.2	28.8	4.38	3, 414	0.005	0.03	2 > 3, 4
Somatization	0.56	0.67	0.77	0.72	0.50	0.70	0.43	0.63	5.44	3, 414	0.001	0.04	2 > 3, 4
Obsessive-compulsive	1.06	0.67	1.27	0.73	1.12	0.63	0.91	0.65	3.93	3, 414	0.009	0.03	2 > 4
Anxiety	0.72	0.52	1.07	0.80	0.86	0.77	0.85	0.62	3.39	3, 414	0.018	0.02	2 > 1
Psychoticism	0.63	0.46	0.89	0.74	0.72	0.67	0.60	0.66	3.41	3, 414	0.018	0.02	ns
GSI	0.81	0.44	1.02	0.64	0.83	0.59	0.76	0.54	4.37	3, 414	0.005	0.03	2 > 3, 4
PST	28.2	11.3	30.6	13.9	26.4	12.3	24.20	13.1	4.53	3, 414	0.004	0.03	2 > 3, 4
PSDI	1.46	0.38	1.67	0.51	1.56	0.50	1.55	0.46	2.63	3, 414	0.050	0.02	2 > 1
SAS													
Total	32.3	8.34	35.2	7.79	33.2	7.39	33.7	5.79	2.83	3, 41	0.038	0.02	ns
Motor	1.60	0.52	1.82	0.58	1.63	0.49	1.63	0.45	4.39	3, 41	0.005	0.03	2 > 3

Notes: BSI - Brief Symptom Inventory; GSI - Global Severity Index; PST - Positive Symptom Total; PSDI - Positive Symptom Distress Index; SAS - Self-Rating Anxiety Scale; ns—not significant; *F* = ANOVA *F* test; *p* = *p*-value; *η*^2^ = Eta squared.

**Table 13:** Significant means differences of dependent variables in relation to daily Internet use.

Variables	Daily Internet Use				*t*-Test			
	No (*n* = 67)		Yes (*n* = 351)					
	*M*	*SD*	*M*	*SD*	*t*	*df*	*p*	*d*
**BDI**								
Total	8.24	7.11	6.13	6.75	2.33	416	0.020	0.31
Cognitive-affective	0.41	0.39	0.27	0.36	2.81	416	0.005	0.38

Notes: BDI - Beck Depression Inventory.

**Table 14:** Significant means differences of dependent variables in relation to hours of Internet use.

Variables	Hours Per Day of Internet Use						One-Way ANOVA				Games–Howell Test
	≤1 (*n* = 137)		2–4 (*n* = 208)		≥5 (*n* = 73)						
	*M*	*SD*	*M*	*SD*	*M*	*SD*	*F*	*df*	*p*	*η^2^*	
**UCLA**											
Total	36.3	9.66	39.4	8.67	40.21	8.68	6.54	2, 415	.002	0.03	2, 3 >1

Notes: UCLA - UCLA Loneliness Scale; *F*= ANOVA *F* test; *p* = *p*-value; *η*^2^ = Eta squared.

**Table 15:** Sample frequencies of dependent variables based on cutoff points.

Cutoff Points	*n*	%
**BSI (PSDI)**		
< 1.7 (without symptoms)	279	66.7
≥ 1.7 (with symptoms)	139	33.3
**BDI**		
0–9 (non-depressed)	310	74.2
10–16 (dysphoria)	67	16.0
17–20 (mild depressive states)	20	4.80
21–30 (moderate depression)	19	4.50
> 30 (severe depression)	2	0.50
**SAS**		
20–44 (normal)	378	90.4
45–59 (mild to moderate anxiety)	37	8.90
60–74 (marked to severe anxiety)	3	0.70
75–80 (extreme anxiety)	0	0.00
**UCLA**		
≤ 45 (without loneliness)	317	75.8
> 45 (with loneliness)	101	24.2

Notes: BSI - Brief Symptom Inventory; PSDI - Positive Symptom Distress Index; BDI - Beck Depression Inventory; SAS - Self-Rating Anxiety Scale; UCLA - UCLA Loneliness Scale.
